# *N*-(2-Ethyl­phen­yl)phthalimide

**DOI:** 10.1107/S1600536808020448

**Published:** 2008-08-06

**Authors:** Yen May Fan, Norzalida Zakaria, Azhar Ariffin, Seik Weng Ng

**Affiliations:** a Department of Chemistry, University of Malaya, 50603 Kuala Lumpur, Malaysia

## Abstract

In the title compound, C_16_H_13_NO_2_, the phthalimide and benzene ring systems form a dihedral angle of 77.2 (1)°.

## Related literature

The crystal structures of a number of phenyl-substituted *N*-phenyl­phthalimides have been reported. For the 2-tolyl analogue, see: Bocelli & Cantoni (1989 ▶). For the 2,4-dimethyl­phenyl analogue, see: Magnomedova *et al.* (1980 ▶); Shahzadi *et al.* (2006 ▶). For the 2,6-dimethyl­phenyl and 2,4,6-trimethyl­phenyl analogues, see: Voliotis *et al.* (1984 ▶). For background literature on kinetic studies, see: Sim *et al.* (2006 ▶, 2007 ▶).
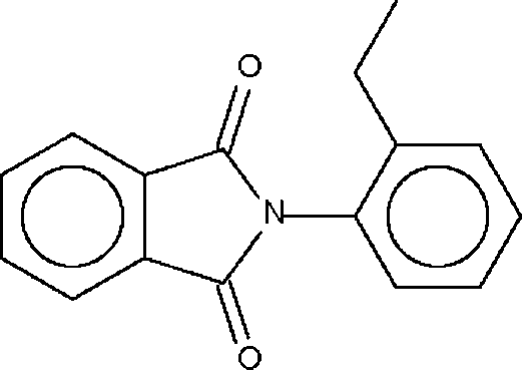


## Experimental

### 

#### Crystal data

C_16_H_13_NO_2_
*M* *_r_* = 251.27
Orthorhombic, 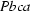

*a* = 15.344 (2) Å
*b* = 7.7731 (8) Å
*c* = 21.693 (2) Å
*V* = 2587.4 (5) Å^3^
*Z* = 8
Mo *K*α radiation
μ = 0.09 mm^−1^
*T* = 100 (2) K
0.15 × 0.10 × 0.05 mm

#### Data collection

Bruker SMART APEX diffractometer
Absorption correction: none
15518 measured reflections
2961 independent reflections
2204 reflections with *I* > 2σ(*I*)
*R*_int_ = 0.054

#### Refinement

*R*[*F* ^2^ > 2σ(*F* ^2^)] = 0.042
*wR*(*F* ^2^) = 0.104
*S* = 1.01
2961 reflections
172 parameters
H-atom parameters constrained
Δρ_max_ = 0.27 e Å^−3^
Δρ_min_ = −0.19 e Å^−3^

### 

Data collection: *APEX2* (Bruker, 2007 ▶); cell refinement: *SAINT* (Bruker, 2007 ▶); data reduction: *SAINT*; program(s) used to solve structure: *SHELXS97* (Sheldrick, 2008 ▶); program(s) used to refine structure: *SHELXL97* (Sheldrick, 2008 ▶); molecular graphics: *X-SEED* (Barbour, 2001 ▶); software used to prepare material for publication: *publCIF* (Westrip, 2008 ▶).

## Supplementary Material

Crystal structure: contains datablocks global, I. DOI: 10.1107/S1600536808020448/tk2280sup1.cif

Structure factors: contains datablocks I. DOI: 10.1107/S1600536808020448/tk2280Isup2.hkl

Additional supplementary materials:  crystallographic information; 3D view; checkCIF report

## Figures and Tables

**Fig. 1.**
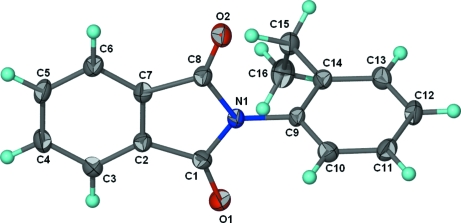
Molecular structure of (I) drawn at the 70% probability level showing atom labelling. Hydrogen atoms are drawn as spheres of arbitrary radiius.

## supplementary crystallographic information

### Comment

In our studies aimed at understanding the nature of intramolecular general base (IGB) and intramolecular general acid (IGA) catalysis in the hydrolysis of *N*-substituted phthalimides, we required the preparation of *N*-phenylphthalimides having a substituent at either the *ortho*- and/or the *para*-position. The title compound (I, Fig. 1) is an such an example. In (I), the phthalimido and phenylene portions are flat and are inclined at an angle of 77.2 (1)°.

### Experimental

Phthalic anhydride (5.0 g, 33.8 mmol) and *o*-ethylaniline (4.91 g, 40.5 mmol) were dissolved in glacial acetic acid (15 ml). The mixture was heated at 393–413 K for 4 h; the completion of the reaction was monitored by thin layer chromatography. The mixture was quenched with water. The solid that separated was collected and recrystallized twice from ethanol to give colorless crystals of (I) in 90% yield.

### Refinement

Carbon-bound H-atoms were placed in calculated positions (C—H 0.95 to 0.99 Å) and were included in the refinement in the riding model approximation, with *U*(H) set to 1.2–1.5*U*(C).

### Crystal data

**Table:**

C_16_H_13_NO_2_	*F*_000_ = 1056
*M**_r_* = 251.27	*D*_x_ = 1.290 Mg m^−^^3^
Orthorhombic, *P**b**c**a*	Mo *K*α radiation λ = 0.71073 Å
Hall symbol: -P 2ac 2ab	Cell parameters from 2189 reflections
*a* = 15.344 (2) Å	θ = 2.3–24.0º
*b* = 7.7731 (8) Å	µ = 0.09 mm^−^^1^
*c* = 21.693 (2) Å	*T* = 100 (2) K
*V* = 2587.4 (5) Å^3^	Irregular block, colourless
*Z* = 8	0.15 × 0.10 × 0.05 mm

### Data collection

**Table:**

Bruker SMART APEX diffractometer	2204 reflections with *I* > 2σ(*I*)
Radiation source: fine-focus sealed tube	*R*_int_ = 0.054
Monochromator: graphite	θ_max_ = 27.5º
*T* = 100(2) K	θ_min_ = 2.3º
ω scans	*h* = −19→11
Absorption correction: none	*k* = −10→10
15518 measured reflections	*l* = −28→28
2961 independent reflections	

### Refinement

**Table:**

Refinement on *F*^2^	Secondary atom site location: difference Fourier map
Least-squares matrix: full	Hydrogen site location: inferred from neighbouring sites
*R*[*F*^2^ > 2σ(*F*^2^)] = 0.042	H-atom parameters constrained
*wR*(*F*^2^) = 0.104	*w* = 1/[σ^2^(*F*_o_^2^) + (0.0466*P*)^2^ + 0.7719*P*] where *P* = (*F*_o_^2^ + 2*F*_c_^2^)/3
*S* = 1.01	(Δ/σ)_max_ = 0.001
2961 reflections	Δρ_max_ = 0.27 e Å^−^^3^
172 parameters	Δρ_min_ = −0.19 e Å^−^^3^
Primary atom site location: structure-invariant direct methods	Extinction correction: none

### Fractional atomic coordinates and isotropic or equivalent isotropic displacement parameters (Å^2^)

**Table:**

	*x*	*y*	*z*	*U*_iso_*/*U*_eq_	
O1	0.48223 (7)	0.23894 (15)	0.51820 (5)	0.0271 (3)	
O2	0.35501 (7)	0.02107 (14)	0.69268 (5)	0.0251 (3)	
N1	0.44007 (8)	0.11973 (15)	0.61157 (5)	0.0171 (3)	
C1	0.42560 (10)	0.19354 (18)	0.55323 (6)	0.0181 (3)	
C2	0.32925 (9)	0.20247 (18)	0.54575 (6)	0.0166 (3)	
C3	0.28005 (10)	0.2658 (2)	0.49751 (7)	0.0204 (3)	
H3	0.3066	0.3137	0.4619	0.024*	
C4	0.18974 (10)	0.2565 (2)	0.50335 (7)	0.0217 (3)	
H4	0.1538	0.2979	0.4709	0.026*	
C5	0.15121 (10)	0.1874 (2)	0.55597 (7)	0.0226 (3)	
H5	0.0895	0.1819	0.5586	0.027*	
C6	0.20135 (10)	0.12648 (19)	0.60476 (7)	0.0203 (3)	
H6	0.1752	0.0812	0.6410	0.024*	
C7	0.29078 (10)	0.13462 (18)	0.59830 (6)	0.0173 (3)	
C8	0.36106 (9)	0.08198 (18)	0.64160 (7)	0.0176 (3)	
C9	0.52461 (9)	0.09843 (18)	0.63950 (6)	0.0162 (3)	
C10	0.57623 (10)	−0.03944 (19)	0.62150 (7)	0.0201 (3)	
H10	0.5564	−0.1168	0.5907	0.024*	
C11	0.65713 (10)	−0.0634 (2)	0.64893 (7)	0.0221 (3)	
H11	0.6929	−0.1572	0.6368	0.027*	
C12	0.68547 (10)	0.0498 (2)	0.69407 (7)	0.0222 (3)	
H12	0.7405	0.0330	0.7132	0.027*	
C13	0.63332 (10)	0.18808 (19)	0.71133 (7)	0.0208 (3)	
H13	0.6535	0.2653	0.7421	0.025*	
C14	0.55168 (10)	0.21587 (18)	0.68431 (6)	0.0174 (3)	
C15	0.49902 (10)	0.37245 (19)	0.70174 (7)	0.0215 (3)	
H15A	0.5072	0.3970	0.7462	0.026*	
H15B	0.4364	0.3489	0.6947	0.026*	
C16	0.52646 (11)	0.5296 (2)	0.66405 (8)	0.0293 (4)	
H16A	0.4912	0.6290	0.6763	0.044*	
H16B	0.5175	0.5061	0.6201	0.044*	
H16C	0.5882	0.5542	0.6716	0.044*	

### Atomic displacement parameters (Å^2^)

**Table:**

	*U*^11^	*U*^22^	*U*^33^	*U*^12^	*U*^13^	*U*^23^
O1	0.0171 (6)	0.0436 (7)	0.0207 (6)	−0.0029 (5)	0.0029 (5)	0.0057 (5)
O2	0.0185 (6)	0.0354 (6)	0.0215 (5)	−0.0017 (5)	−0.0006 (5)	0.0095 (5)
N1	0.0125 (6)	0.0226 (6)	0.0161 (6)	−0.0005 (5)	−0.0006 (5)	0.0014 (5)
C1	0.0171 (8)	0.0212 (7)	0.0160 (7)	−0.0012 (6)	−0.0001 (6)	−0.0013 (6)
C2	0.0144 (7)	0.0177 (7)	0.0176 (7)	−0.0006 (6)	−0.0005 (6)	−0.0015 (5)
C3	0.0192 (8)	0.0248 (8)	0.0173 (7)	0.0003 (6)	−0.0008 (6)	0.0004 (6)
C4	0.0195 (8)	0.0238 (8)	0.0217 (7)	0.0036 (6)	−0.0062 (6)	0.0004 (6)
C5	0.0117 (7)	0.0279 (8)	0.0283 (8)	0.0010 (6)	−0.0013 (6)	−0.0007 (7)
C6	0.0157 (8)	0.0238 (8)	0.0215 (7)	−0.0013 (6)	0.0015 (6)	0.0021 (6)
C7	0.0163 (7)	0.0173 (7)	0.0183 (7)	0.0009 (6)	−0.0012 (6)	0.0001 (6)
C8	0.0148 (7)	0.0191 (7)	0.0191 (7)	−0.0006 (6)	−0.0005 (6)	−0.0004 (6)
C9	0.0106 (7)	0.0216 (7)	0.0164 (7)	−0.0016 (6)	0.0002 (6)	0.0035 (5)
C10	0.0193 (8)	0.0222 (8)	0.0188 (7)	−0.0014 (6)	−0.0007 (6)	−0.0025 (6)
C11	0.0177 (8)	0.0236 (8)	0.0251 (8)	0.0048 (6)	0.0018 (6)	0.0001 (6)
C12	0.0146 (8)	0.0265 (8)	0.0254 (8)	−0.0003 (6)	−0.0032 (6)	0.0029 (6)
C13	0.0173 (8)	0.0240 (8)	0.0211 (7)	−0.0041 (6)	−0.0029 (6)	−0.0009 (6)
C14	0.0149 (7)	0.0195 (7)	0.0179 (7)	−0.0010 (6)	0.0007 (6)	0.0013 (6)
C15	0.0189 (8)	0.0221 (8)	0.0234 (7)	0.0017 (6)	−0.0025 (6)	−0.0032 (6)
C16	0.0270 (9)	0.0239 (8)	0.0369 (9)	0.0032 (7)	−0.0026 (7)	0.0028 (7)

### Geometric parameters (Å, °)

**Table:**

O1—C1	1.2072 (17)	C9—C10	1.389 (2)
O2—C8	1.2085 (17)	C9—C14	1.397 (2)
N1—C1	1.4071 (18)	C10—C11	1.389 (2)
N1—C8	1.4072 (18)	C10—H10	0.9500
N1—C9	1.4412 (18)	C11—C12	1.387 (2)
C1—C2	1.489 (2)	C11—H11	0.9500
C2—C3	1.381 (2)	C12—C13	1.391 (2)
C2—C7	1.3879 (19)	C12—H12	0.9500
C3—C4	1.393 (2)	C13—C14	1.400 (2)
C3—H3	0.9500	C13—H13	0.9500
C4—C5	1.393 (2)	C14—C15	1.509 (2)
C4—H4	0.9500	C15—C16	1.529 (2)
C5—C6	1.392 (2)	C15—H15A	0.9900
C5—H5	0.9500	C15—H15B	0.9900
C6—C7	1.381 (2)	C16—H16A	0.9800
C6—H6	0.9500	C16—H16B	0.9800
C7—C8	1.488 (2)	C16—H16C	0.9800
			
C1—N1—C8	111.43 (12)	C14—C9—N1	119.02 (13)
C1—N1—C9	124.54 (12)	C9—C10—C11	119.53 (14)
C8—N1—C9	123.84 (11)	C9—C10—H10	120.2
O1—C1—N1	124.87 (14)	C11—C10—H10	120.2
O1—C1—C2	129.23 (13)	C12—C11—C10	119.83 (14)
N1—C1—C2	105.90 (12)	C12—C11—H11	120.1
C3—C2—C7	121.70 (14)	C10—C11—H11	120.1
C3—C2—C1	129.94 (13)	C11—C12—C13	120.01 (14)
C7—C2—C1	108.36 (12)	C11—C12—H12	120.0
C2—C3—C4	117.13 (14)	C13—C12—H12	120.0
C2—C3—H3	121.4	C12—C13—C14	121.41 (14)
C4—C3—H3	121.4	C12—C13—H13	119.3
C5—C4—C3	121.11 (14)	C14—C13—H13	119.3
C5—C4—H4	119.4	C9—C14—C13	117.17 (13)
C3—C4—H4	119.4	C9—C14—C15	122.84 (13)
C4—C5—C6	121.31 (14)	C13—C14—C15	119.91 (13)
C4—C5—H5	119.3	C14—C15—C16	111.27 (13)
C6—C5—H5	119.3	C14—C15—H15A	109.4
C7—C6—C5	117.18 (14)	C16—C15—H15A	109.4
C7—C6—H6	121.4	C14—C15—H15B	109.4
C5—C6—H6	121.4	C16—C15—H15B	109.4
C6—C7—C2	121.56 (13)	H15A—C15—H15B	108.0
C6—C7—C8	130.07 (13)	C15—C16—H16A	109.5
C2—C7—C8	108.35 (13)	C15—C16—H16B	109.5
O2—C8—N1	124.92 (13)	H16A—C16—H16B	109.5
O2—C8—C7	129.13 (13)	C15—C16—H16C	109.5
N1—C8—C7	105.95 (12)	H16A—C16—H16C	109.5
C10—C9—C14	122.04 (13)	H16B—C16—H16C	109.5
C10—C9—N1	118.94 (13)		
			
C8—N1—C1—O1	179.48 (14)	C9—N1—C8—C7	175.82 (12)
C9—N1—C1—O1	4.4 (2)	C6—C7—C8—O2	−0.6 (3)
C8—N1—C1—C2	−0.23 (15)	C2—C7—C8—O2	178.26 (15)
C9—N1—C1—C2	−175.35 (12)	C6—C7—C8—N1	−179.73 (15)
O1—C1—C2—C3	−0.7 (3)	C2—C7—C8—N1	−0.86 (15)
N1—C1—C2—C3	179.03 (14)	C1—N1—C9—C10	−80.22 (18)
O1—C1—C2—C7	179.98 (15)	C8—N1—C9—C10	105.25 (16)
N1—C1—C2—C7	−0.33 (15)	C1—N1—C9—C14	100.51 (16)
C7—C2—C3—C4	−0.9 (2)	C8—N1—C9—C14	−74.03 (18)
C1—C2—C3—C4	179.78 (14)	C14—C9—C10—C11	0.5 (2)
C2—C3—C4—C5	0.5 (2)	N1—C9—C10—C11	−178.73 (13)
C3—C4—C5—C6	0.5 (2)	C9—C10—C11—C12	0.2 (2)
C4—C5—C6—C7	−1.1 (2)	C10—C11—C12—C13	−0.7 (2)
C5—C6—C7—C2	0.7 (2)	C11—C12—C13—C14	0.4 (2)
C5—C6—C7—C8	179.48 (14)	C10—C9—C14—C13	−0.8 (2)
C3—C2—C7—C6	0.3 (2)	N1—C9—C14—C13	178.48 (12)
C1—C2—C7—C6	179.72 (13)	C10—C9—C14—C15	176.18 (13)
C3—C2—C7—C8	−178.69 (13)	N1—C9—C14—C15	−4.6 (2)
C1—C2—C7—C8	0.73 (15)	C12—C13—C14—C9	0.3 (2)
C1—N1—C8—O2	−178.51 (14)	C12—C13—C14—C15	−176.74 (14)
C9—N1—C8—O2	−3.3 (2)	C9—C14—C15—C16	−92.56 (17)
C1—N1—C8—C7	0.66 (15)	C13—C14—C15—C16	84.31 (17)
